# Characterization of Aspirated Duodenal Fluids from Parkinson’s Disease Patients

**DOI:** 10.3390/pharmaceutics15041243

**Published:** 2023-04-14

**Authors:** Tom de Waal, Joachim Brouwers, Philippe Berben, Talia Flanagan, Jan Tack, Wim Vandenberghe, Tim Vanuytsel, Patrick Augustijns

**Affiliations:** 1 Drug Delivery and Disposition, KU Leuven, 3000 Leuven, Belgium; 2 Pharmaceutical Sciences, UCB Pharma SA, 1420 Braine-l’Alleud, Belgium; 3 Department of Gastroenterology and Hepatology, University Hospitals Leuven, 3000 Leuven, Belgium; 4 Translational Research Center for Gastrointestinal Disorders, TARGID, KU Leuven, 3000 Leuven, Belgium; 5 Department of Neurology, University Hospitals Leuven, 3000 Leuven, Belgium

**Keywords:** Parkinson’s disease, duodenal fluid composition, drug absorption, gastrointestinal fluid aspiration, clinical study

## Abstract

Parkinson’s disease, one of the most common neurodegenerative diseases, may not only affect the motor system, but also the physiology of the gastrointestinal tract. Delayed gastric emptying, impaired motility and altered intestinal bacteria are well-established consequences of the disease, which can have a pronounced effect on the absorption of orally administered drugs. In contrast, no studies have been performed into the composition of intestinal fluids. It is not unlikely that Parkinson’s disease also affects the composition of intestinal fluids, a critical factor in the in vitro and in silico simulation of drug dissolution, solubilization and absorption. In the current study, duodenal fluids were aspirated from Parkinson’s disease (PD) patients and age-matched healthy controls (healthy controls, HC) consecutively in fasted and fed conditions. The fluids were then characterized for pH, buffer capacity, osmolality, total protein, phospholipids, bile salts, cholesterol and lipids. In a fasted state, the intestinal fluid composition was highly similar in PD patients and healthy controls. In general, the same was true for fed-state fluids, apart from a slightly slower and less pronounced initial change in factors directly affected by the meal (i.e., buffer capacity, osmolality, total protein and lipids) in PD patients. The absence of a fast initial increase for these factors immediately after meal intake, as was observed in healthy controls, might result from slower gastric emptying in PD patients. Irrespective of the prandial state, a higher relative amount of secondary bile salts was observed in PD patients, potentially indicating altered intestinal bacterial metabolism. Overall, the data from this study indicate that only minor disease-specific adjustments in small intestinal fluid composition should be considered when simulating intestinal drug absorption in PD patients.

## 1. Introduction

Parkinson’s disease (PD) is one of the most common neurodegenerative diseases and is hallmarked by motor symptoms, including rigidity, bradykinesia and tremor caused by a loss of dopaminergic neurons in the brain. In the past decades, non-motor symptoms, especially gastrointestinal (GI) symptoms, have gathered interest as potential early markers for PD [1,2]. In some studies, the GI tract has even been postulated to be involved in the etiology of the disease [3,4]. Non-motor symptoms, including early satiety, bloating and constipation, are often observed before the onset of motor symptoms. Research exploring these symptoms found alterations in the GI physiology of PD patients, including reduced salivation, impaired motility and microbiota dysregulation [5].

The changes in GI physiology do not only affect the patients’ quality of life, but can also have a pronounced effect on the performance of orally administered drugs and, thus, treatment outcome [6,7,8]. The best documented example is levodopa, the primary treatment for PD. PD-induced gastroparesis may cause inconsistent absorption of levodopa, which contributes to fluctuations in motor symptoms that occur in more than half of the patients within five years of levodopa treatment [9]. This implies that it is of utmost importance to understand all potential GI modifications in PD patients. Whereas there exists a reasonable dataset for some GI physiological parameters, other critical factors have been systematically overlooked, including GI fluid volume and its composition [5,10]. The GI fluid composition can significantly affect drug solubility and dissolution in the GI lumen. For instance, bile salts, phospholipids and lipid digestion products are the main building blocks of colloidal structures (e.g., micelles, vesicles and lipid droplets) that have the ability to solubilize lipophilic drugs. The pH and buffer capacity of GI fluids may substantially affect the solubility and dissolution of ionizable drugs [11,12,13]. For this reason, simulated intestinal fluids (SIFs) based on the composition of human intestinal fluids from heathy adults have been developed to improve the predictive value of in vitro solubility and dissolution tools [12,14]. These SIFs contain physiologically relevant levels of pH, buffer capacity, bile salts (i.e., taurocholic acid) and phospholipids (phosphatidylcholine) that reflect the in vivo GI environment in fasted-state conditions. In addition, specific media have been established to simulate the composition of GI fluids in post-prandial conditions. Despite significant progress in understanding the GI environment in healthy young adults, limited information is available on the quantitative and/or qualitative GI fluid composition in patient populations, including among PD patients. As a result, the applicability of the available SIFs to predict drug absorption in PD patients can be questioned. Indeed, it is not unlikely that bile and pancreatic secretions are affected in PD patients in a similar way as their salivary secretion and GI motility (i.e., reduced secretion and contractility), possibly leading to altered drug solubility and dissolution. 

This study aims to characterize the composition of upper small intestinal fluids relevant to drug solubility and dissolution in PD patients and explore differences compared with age-matched healthy controls (HC). Duodenal fluids collected in the fasted state and after a standardized meal (i.e., fed state) were characterized for several factors, including pH, buffer capacity, osmolality, concentrations of total protein, phospholipids, bile salts, cholesterol and lipid digestion products. Insights into the intestinal fluid composition in PD patients would allow the tailoring of in vitro and in silico tools for the prediction of intestinal drug absorption to this specific population.

## 2. Materials and Methods

### 2.1. Materials

Taurochenodeoxycholic acid (TCDC), taurodeoxycholic acid (TDC), glycoursodeoxycholic acid (GUDC), glycochenodeoxycholic acid (GCDC), glycodeoxycholic acid (GDC), glycocholic acid (GC), chenodeoxycholic acid (CDC), deoxycholic acid (DC), lithocholic acid (LC), cholic acid (C), sodium hydroxide (NaOH), cholesterol (Chol), cholesteryl oleate, cholesteryl palmitate (Cholp), tripalmitin (TP), triolein, trilinolein, dipalmitin, diolein, dilinolein (DL), monooleate (MO), monopalmitin, monolinolein, palmitic acid, oleic acid (OA), linoleic acid, 1-octadecanol, decyl oleate, urea, trisaminomethane, orlistat and L-tryptophan were purchased from Sigma-Aldrich (St. Louis, MO). Tauroursodeoxycholic acid (TUDC), ursodeoxycholic acid (UDC), and taurocholic acid (TC) were acquired from Calbiochem (Darmstadt, Germany). Deuterated cholic acid (C-d4) was purchased from Cayman Chemical (Ann Arbor, MI, USA) and deuterated chenodeoxycholic acid (CDC-d4) from Alsachim (Illkirch Graffenstaden, France). Hydrochloric acid (HCl) and acetonitrile (ACN, HPLC gradient grade) were purchased from Fisher scientific (Waltham, MA, USA) and methanol (MeOH, HPLC grade) from Acros Organics (Waltham, MA, USA). Iso-octane (UV spectroscopy grade), ethyl acetate (LC-MS grade) and acetone (LC-MS grade) were purchased from Carl Roth (Karlsruhe, Germany). Ultrapure water was obtained with an ELGA purifying system (Woodridge, IL, USA).

### 2.2. Patient Selection

PD patients (<80 years) selected for the aspiration study were diagnosed according to the UK Parkinson’s Disease Society Brain Bank criteria [15]. HC were recruited afterwards within the same age range as the PD patients. PD patients were recruited via a flyer distributed through the Flemish PD patient organization (Vlaamse Parkinson Liga); HC were recruited via flyers distributed in the University Hospitals Leuven. The following exclusion criteria were used for PD patients and HC: pregnancy, soy or milk allergies, GI pathologies or previous surgery (e.g., Crohn’s disease, ulcerative colitis, gastric bypass or cholecystectomy), intake of medication altering GI physiology (e.g., proton-pump inhibitors (PPI), antacids, spasmolytics, anti-diarrhea medication, laxatives and anti-emetics) and significant cognitive impairment.

### 2.3. Study Protocol

**Figure 1:**
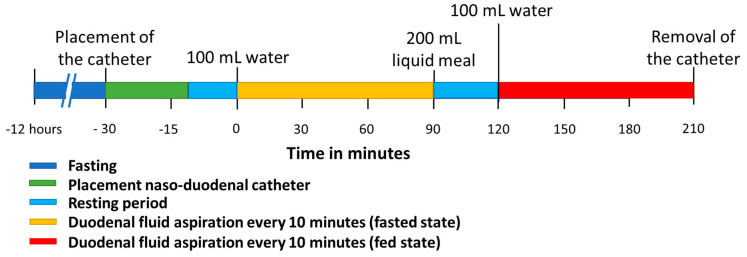
Timeline of the clinical study protocol to aspirate duodenal fluids in fasted and fed state.

**Figure 2:**
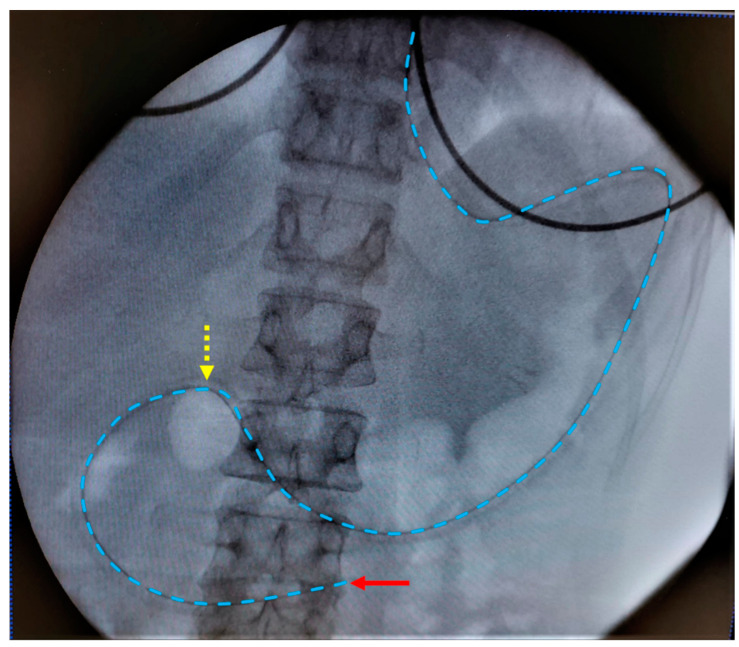
Typical fluoroscopy image with placement of the aspiration tip (red arrow) of the duodenal catheter near the ligament of Treitz. The catheter is indicated by the blue dotted line. The yellow dotted arrow indicates the transition from stomach to duodenum.

The clinical study was approved by the Ethics Committee Research UZ/KU Leuven in Belgium (S63435). All participants gave written informed consent. The study followed a similar protocol as previously applied for healthy volunteers by Riethorst et al. [16]. Figure 1 depicts the study timeline. The participants were instructed to fast for 12 h preceding the placement of the naso-duodenal catheter. During the fasting period, no foods or drinks were allowed except for water and medication. On the morning of the study, water intake was limited to the volume needed to take medication. The duodenal catheter was placed by experienced nurses at the Endoscopy Unit of the University Hospitals Leuven. Local anesthesia of the nose and throat was applied by instilling a 2% lidocaine gel through the nose before placement of the catheter. The aspiration tip of the catheter was placed in the proximity of the ligament of Treitz in the duodenum, which was verified with fluoroscopy. Figure 2 illustrates a typical fluoroscopy image of the catheter placement. After catheter placement, the participant sat upright (Fowler’s position) in a bed for the entire duration of the study. Following a 10 to 20 min acclimatization period, the participant was asked to drink 100 mL of tap water. Up to 5 mL duodenal fluid was aspirated every 10 min for 90 min through the catheter by means of a syringe (fasted state). Following the fasted state, the participant received 200 mL of a liquid meal (Ensure Plus vanilla, Abbott, Chicago, IL, USA) followed by a 30 min waiting period. Before aspiration, the participant ingested another 100 mL of tap water. Duodenal fluids were again aspirated every 10 min for 90 min (fed state) after which the catheter was removed. The water volume administered before each sampling period (100 mL) differed from the recommended 240 mL for the ingestion of tablets and capsules that was used in previous studies to accommodate for the reduced food and drink intake in this patient population, as PD patients often suffer from early satiety and bloating. Similarly, the volume of the liquid meal was reduced to 200 mL instead of the 400 mL that is commonly used to simulate the caloric content of the FDA standardized breakfast.

At each sampling timepoint, the aspirated fluid was aliquoted for the determination of different compositional factors (i.e., pH (100 µL), buffer capacity (400 µL), osmolality (300 µL), bile salts (50 µL), phospholipids (50 µL), total protein (20 µL) and lipid digestion products and cholesterol (200 µL)). The pH and buffer capacity were measured instantaneously, and the remaining aliquots were frozen on dry ice during the remainder of the study and stored at −20 °C afterwards while awaiting further analysis. To minimize ex vivo lipid digestion, lipase activity in the aliquots for determination of lipids and phospholipids was inhibited by adding a 1 mM orlistat stock solution in methanol (final concentration 1 µM). Whenever insufficient intestinal fluid could be aspirated for a full analysis, characteristics were prioritized as follows (considering relevance for drug absorption and required volume for analysis): pH > bile salts > lipid digestion products and cholesterol > phospholipids > total protein > osmolality > buffer capacity.

### 2.4. pH and Buffer Capacity

The pH and buffer capacity were measured immediately after aspiration with a glass electrode pH probe (Hamilton Biotrode) that was calibrated before use. In fasted-state fluids, the acidic and basic buffer capacity was measured in separate aliquots of 200 µL duodenal fluid by the addition of 2 µL of 0.1 M HCl and NaOH, respectively; for fed-state fluids, 2 µL of 1 M HCl and NaOH were added. Since vortexing or vigorous mixing of intestinal fluids may result in increased pH due to the unstable bicarbonate buffer and subsequent evaporation of CO_2_ (more pronounced in fasted-state fluids), an oil layer (150 µL decyl oleate) was added to the aliquots, reducing the extent of evaporation. In addition, the acid or base spikes were mixed gently with the duodenal fluid using the electrode probe. Different concentrations of acid or base were used in fasted- and fed-state duodenal fluids to anticipate the vastly different buffer capacity between both prandial states [17]. The concentrations of the spikes were selected to increase or reduce pH by no more than 1 pH unit per spike. Because the buffer capacity after acid or base addition was almost identical, the average of the two methodologies is reported in this paper.

### 2.5. Osmolality

Osmolality was measured using a freeze point depression osmometer (Advanced Instruments 3250, Norwood, MA, USA). The osmometer was operated in the low-range mode with the buzz point set to 3500. The system suitability was verified by measuring standard solutions of 100, 290 and 1500 mOsm/kg before use.

### 2.6. Total Protein

Total protein concentration was determined using the tryptophan fluorescence assay described by Wiśniewski et al. (2015) [18]. In short, 2 µL of intestinal fluid was added to 200 µL 8 M urea in 100 mM Tris pH 7.8. Fluorescence (excitation 295 nm, emission 350 nm) was measured and compared to a calibration curve of different tryptophan concentrations. Total protein content was calculated assuming an average relative tryptophan content of 1.17% [18].

### 2.7. Phospholipids

Phospholipid concentration was determined using the Labassay Phospholipid kit (Fujifilm, Tokyo, Japan). Phospholipids containing choline are hydrolyzed to choline by phospholipase D. The free choline is subsequently oxidized by choline oxidase producing hydrogen peroxide, which in turn reacts to N-ethyl-N-(2-hydroxy-3-sulfopropyl)-3,5-dimethoxyaniline (DAOS) and 4-aminoantipyrine, resulting in a blue color. The concentration of phospholipids is then quantified by measuring the absorbance (600 nm) using a Tecan infinite m200 plate reader (Tecan, Männedorf, Switzerland).

Only choline-containing phospholipids (i.e., phosphatidylcholine, lysophosphatidylcholine and sphingomyelin) are detected with this method. In fasted-state fluids, around 98% of the total phospholipids consist of lysophosphatidylcholine and phosphatidylcholine [19]; in fed-state fluids, other dietary non-choline phospholipids may be present, albeit to a lesser extent than choline-based phospholipids.

Although the liquid meal used in the present study also contains free choline that will react with the color reagent, the possible bias is likely limited considering the concentration of choline chloride in the meal only amounts to around 0.7 mM, which is further diluted after ingestion by stomach fluids and the co-administered glass of water.

### 2.8. Bile Salts

Bile salts were measured following an adapted method from Riethorst et al. [16] Bile salts were separated on an Acquity UPLC H-class system (Waters) equipped with a Kinetex XB-C18 column (2.6 µm 50 × 2.1 mm, Phenomenex) via gradient elution. The column was kept at 35 °C and 5 µL of sample was injected. Detection was performed using a Xevo TSQ micro triple quadrupole mass spectrometer (Waters) with electrospray ionization in both positive and negative modes. The LC gradient, mass spectrometry settings and individual bile salt mass transitions are described in Supplementary Tables S1 and S2, respectively. Aspirated duodenal fluids were diluted 1000 and 10,000 times in 50/50 MeOH–water containing 200 nM of both C-d4 and CDC-d4 as internal standards before analysis. During each run, analytical accuracy and precision were monitored using quality control (QC) samples (20, 150 and 1250 nM of all bile salts); the accuracy remained between 90 and 110% and relative standard deviations were below 5%.

### 2.9. Lipid Digestion Products and Cholesterol

Lipids (i.e., triacylglycerides (TAGs), diacylglycerides (DAGs), monoacylglycerides (MAGs) and free fatty acids (FFAs)) and cholesterol (i.e., Chol and CholE) were determined with high-performance liquid chromatography (HPLC) coupled to a charged aerosol detector (CAD) using a method adapted from Infantes-Garcia et al. [20].

Lipids were extracted from 100 µL of intestinal fluid, to which 10 µL 4 mg/mL 1-octadecanol (internal standard) in chloroform–isooctane (50:50) and 100 µL of 1M HCl were added. After mixing, 200 µL of extraction medium consisting of chloroform and isooctane in a 1:1 ratio was added. Lipids and cholesterol were extracted by 3 cycles of 20 s of vigorous shaking before vortexing for 1 min. After centrifugation (20.000 *g* for 15 min at 4 °C), the organic bottom layer was collected and diluted 1:5 in pure isooctane before injecting 1 µL into the HPLC system.

Separation was performed using a Vanquish dual pump system (Thermo Fisher Scientific, Waltham, MA) equipped with an Ascentis Express OH5 column (100 × 2.1 mm, 2.7 µm) kept at 35 °C. Lipids and cholesterol products were eluted per lipid class using a gradient specified in Supplementary Table S3. The mobile phase composition at the detector was kept stable by employing a secondary pump with an inversed gradient to the analytical gradient to ensure a more uniform analyte response for all analytes. The flow rate was 0.5 mL/min for each of the pumps (i.e., 1 mL/min at the detector). The CAD evaporation temperature was set to 35 °C, data collection at 10 Hz, filter constant to 3.6 and the power function between 1.2 and 1.6, depending on the lipid class. For quantification, a calibration curve was prepared using one surrogate reference product per lipid class (i.e., TAGs, DAGs, MAGs, FFA, Chol, CholE) as specified in Supplementary Table S4.

The method was validated by spiking known concentrations of reference lipids for all classes (i.e., lipids derived from palmitic, oleic or linoleic acid) in simulated fasted- and fed-state intestinal fluids (Fasted State Simulated Intestinal Fluid, FaSSIF and Fed State Simulated Intestinal Fluid, FeSSIF; Biorelevant, London, UK). The average accuracy and precision of three separate runs at different concentrations are given in Supplementary Table S5. In addition, recovery in human intestinal fluids was evaluated by spiking a known concentration of one reference product per lipid class in six different pools of human intestinal fluid (e.g., fasted- and fed-state human intestinal fluids). During each run, analytical accuracy and precision were monitored using QC samples (0.25, 0.063 and 0.016 mg/mL for FFA and 0.063, 0.016 and 0.004 mg/mL for all other lipid classes); the accuracy was between 85 and 115% and relative standard deviations were below 10%.

## 3. Results

### 3.1. Study Subjects

Naso-duodenal intubation and duodenal fluid aspiration was performed in ten PD patients and nine age-matched healthy controls. In one PD patient, the catheter moved back into the stomach during the sample collection in the fed state; for this reason, all data from this patient were omitted from the current discussion. The PD patients (6 males, 3 females) had an average age of 67.0 ± 9.9 years (mean ± SD) and a median Hoehn–Yahr score ON medication of 2 (range 1–3) [21], and scored a median of 5 (range 3–10) on the GI section (question 1–7) of the Scales for Outcomes in Parkinson’s Disease—Autonomic Dysfunction (SCOPA-AUT GI) [22] questionnaire. The healthy controls (4 males, 5 females) had an age of 64.3 ± 8.5 years (no statistical difference between the ages of the PD patients and HC, *p* = 0.55, unpaired *t*-test) and scored 0 (range 0–1) on the SCOPA-AUT GI. The SCOPA-AUT GI score is a measure of the severity of GI symptoms in patients and healthy controls; the maximum SCOPA-AUT GI score amounts to 21 and indicates severe GI symptoms, including frequent drooling, chocking, bloating, constipation and involuntary stool loss. An overview of the individual demographic data is listed in Table 1. In addition to disease-specific medication, the PD patients were treated for heart problems (i.e., high blood pressure) (6/9), high cholesterol (4/9), prostate problems (2/9), allergy (1/9), pain (2/9) and insomnia (4/9). The healthy controls were treated for asthma/allergy (2/9), high blood pressure (2/9) and prostate problems (2/9). Four healthy controls did not take any medication. While the recruitment of study participants excluded PD patients and healthy controls taking medication which directly alters GI physiology, the effects of other drugs, including PD-specific medication or opioids, cannot be ruled out. Such confounding factors are inherent to the study population, considering the generally older age and the common prevalence of comorbidities.

All patients fasted for at least 12 h before the intubation. Duodenal fluids were collected every 10 min for 90 min in both fasted state (after intake of 100 mL tap water) and fed state (after intake of 200 mL liquid meal and 100 mL tap water), resulting in 20 fluid samples per patient. At some timepoints, no or only a limited volume of intestinal fluid could be aspirated; in the latter case, samples were only partly analyzed according to the priority specified in Section 2.3 (Study protocol). Overall, 236 intestinal fluid samples were fully characterized (113 from PD patients, 123 from healthy controls) and another 112 samples were partly characterized (57 from PD patients, 55 from healthy controls). Twelve samples (10 from PD patients, 2 from healthy controls) could not be aspirated.

**Figure 3:**
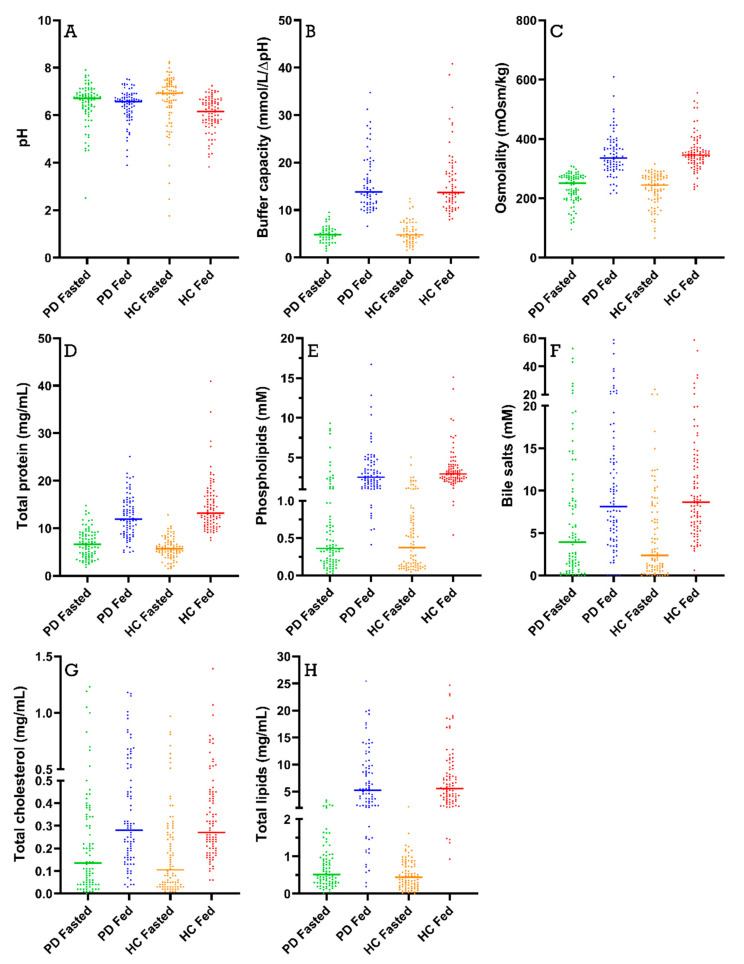
Scatter plots for (**A**) pH; (**B**) buffer capacity; (**C**) osmolality; (**D**) total protein concentration; (**E**) phospholipid concentration; (**F**) total bile salt concentration; (**G**) total cholesterol concentration; and (**H**) total lipid concentration. Each data point represents a single duodenal fluid sample collected from Parkinson’s disease patients (PD) or healthy controls (HC) in either a fasted- or fed-state condition; the lines represent the mean of the group.

Figure 3 depicts the scatterplots and mean values for the different fluid characteristics (i.e., pH, buffer capacity, osmolality, total protein, phospholipids, total bile salts, total lipids and total cholesterol) measured in all duodenal samples grouped by population and prandial state, independently of sampling time. Time profiles for the different characteristics are presented in Section 3.2, Section 3.3, Section 3.4, Section 3.5, Section 3.6 and Section 3.7.

### 3.2. pH and Buffer Capacity

**Figure 4:**
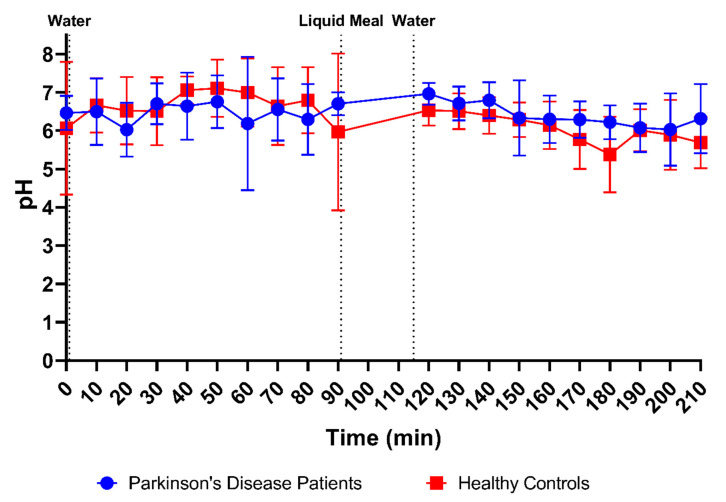
Time-dependent evolution of the pH in duodenal fluids aspirated from Parkinson’s disease patients (*n* = 9, blue circles) and healthy controls (*n* = 9, red squares). Data points represent the mean ± standard deviation; vertical dotted lines indicate the ingestion of 100 mL of water or 200 mL of liquid meal.

Duodenal pH was measured immediately after aspiration in 170 samples from PD patients (85 fasted and 85 fed) and in 178 samples from healthy controls (88 fasted and 90 fed). As depicted in Figure 3A, the average pH of all duodenal fluid samples aspirated in fasted state was 6.49 ± 0.85 in PD patients and 6.63 ± 1.13 in healthy controls. In the fed state, the average pH of the fluids remained similar in PD patients (6.41 ± 0.69) and decreased slightly in healthy controls (6.06 ± 0.72). Although a decrease in pH after intake of the meal was observed in most participants (14 out of 18), this effect was not seen in the average time-dependent evolution of the pH during the sampling period (Figure 4) due to substantial intersubject variability.

**Figure 5:**
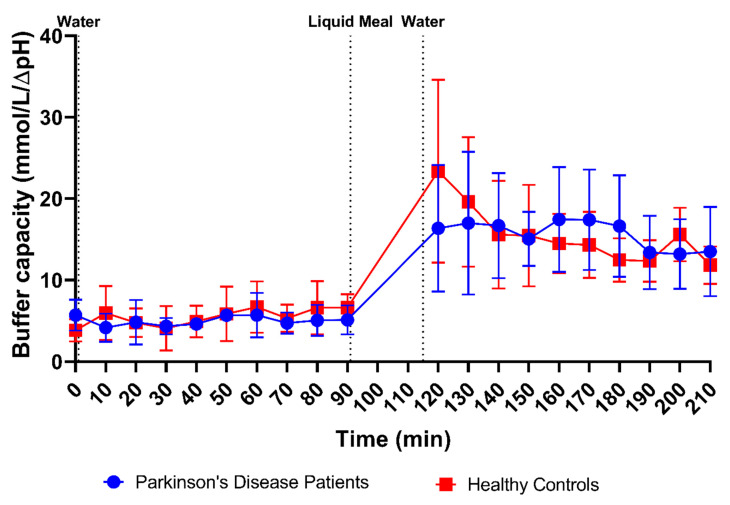
Time-dependent evolution of the buffer capacity in duodenal fluids aspirated from Parkinson’s disease patients (*n* = 9, blue circles) and healthy controls (*n* = 9, red squares). Data points represent the mean ± standard deviation; vertical dotted lines indicate the ingestion of 100 mL of water or 200 mL of liquid meal.

In addition to pH, the buffer capacity was measured immediately after aspiration of the duodenal fluids, as the average of the buffer capacities after acidification and alkalization. Since these measurements used a relatively large amount of fluid, buffer capacity was determined in only 113 samples from PD patients (47 fasted and 66 fed) and 123 samples from healthy controls (55 fasted and 68 fed). As shown in Figure 3B, the average buffer capacity in fasted-state fluids was similar between PD patients and healthy controls (4.90 ± 1.75 mmol/L/ΔpH and 5.30 ± 2.48 mmol/L/ΔpH, respectively) and increased 3-fold in both study populations in fed-state fluids (15.70 ± 5.97 mmol/L/ΔpH and 15.93 ± 6.73 mmol/L/ΔpH, respectively). This meal-induced increase in average buffer capacity was also obvious from the time-dependent evolution depicted in Figure 5. Despite the increase being largely similar in both study populations, a temporary peak in average buffer capacity at the first postprandial sampling point (i.e., 30 min after intake of the meal) was observed in healthy controls, but not in PD patients. This suggests that the initial meal-induced change in buffer capacity was less pronounced in PD patients.

### 3.3. Osmolality

**Figure 6:**
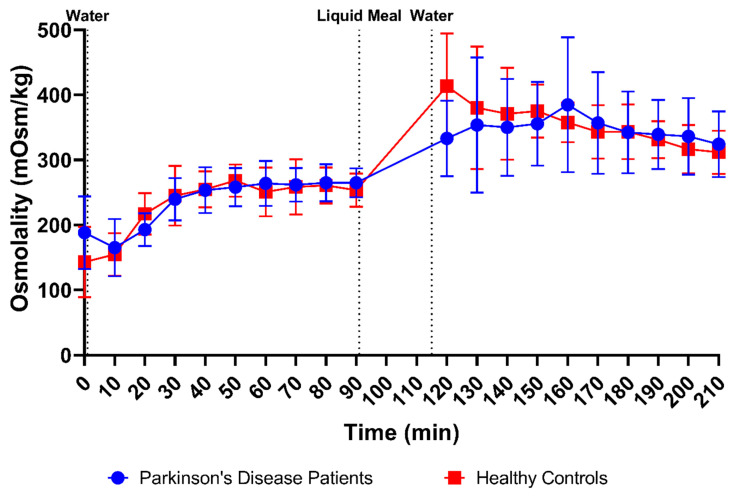
Time-dependent evolution of the osmolality in duodenal fluids aspirated from Parkinson’s disease patients (*n* = 9, blue circles) and healthy controls (*n* = 9, red squares). Data points represent the mean ± standard deviation; vertical dotted lines indicate the ingestion of 100 mL of water or 200 mL of liquid meal.

Osmolality was measured in 166 samples from PD patients (83 fasted and 83 fed) and 176 samples from healthy controls (86 fasted and 90 fed). The average fasted-state osmolality, displayed in Figure 3C, did not differ between PD patients (234 ± 50 mOsm/kg) and healthy controls (231 ± 55 mOsm/kg). In fed-state fluids, the average osmolality increased to a similar extent in PD patients (347 ± 70 mOsm/kg) and healthy controls (354 ± 60 mOsm/kg). As seen in the time-based evolution depicted in Figure 6, the lowest fasted-state osmolality was measured immediately after intake of the glass of water in the fasted state due to dilution of the intestinal contents. Osmolality recovered within the first 40 min and remained stable for the remainder of the fasted-state sampling period. Following ingestion of the meal, the increase in osmolality was clearly seen in both PD patients and healthy controls. Even though the average osmolality–time profiles were highly similar between both study populations, the initial meal-induced rise in osmolality seemed to be more pronounced in healthy controls compared with PD patients.

### 3.4. Total Protein

**Figure 7:**
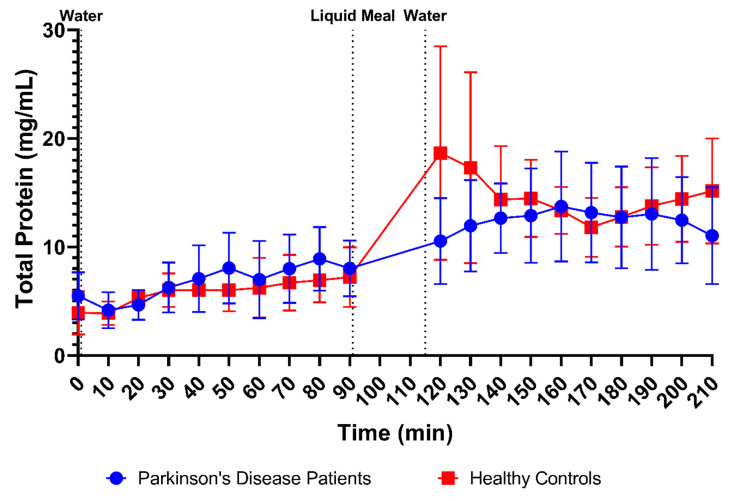
Time-dependent evolution of the total protein concentration in duodenal fluids aspirated from Parkinson’s disease patients (*n* = 9, blue circles) and healthy controls (*n* = 9, red squares). Data points represent the mean ± standard deviation; vertical dotted lines indicate the ingestion of 100 mL of water or 200 mL of liquid meal.

The total protein concentrations were measured in 170 samples from PD patients (85 fasted and 85 fed) and 176 samples from healthy controls (87 fasted and 90 fed). From Figure 3D, it is obvious that the average protein concentrations were comparable between PD patients and healthy controls in fasted-state fluids (6.74 ± 2.96 mg/mL versus 5.79 ± 2.13 mg/mL, respectively) and fed-state fluids (12.41 ± 4.26 mg/mL versus 14.60 ± 5.41 mg/mL, respectively). In addition, the time-dependent evolution of the total protein concentration, depicted in Figure 7, was fairly similar between both study populations. Only the initial meal-induced increase in protein concentration was more pronounced in healthy controls compared with PD patients.

### 3.5. Phospholipids

**Figure 8:**
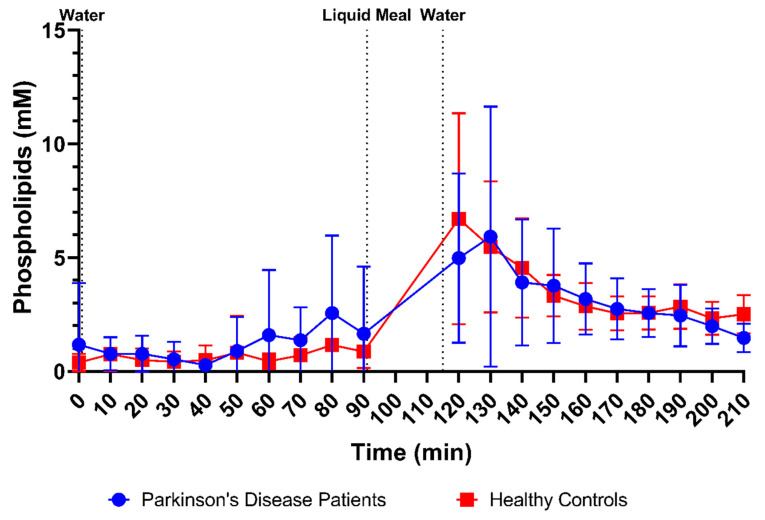
Time-dependent evolution of the phospholipid concentration in duodenal fluids aspirated from Parkinson’s disease patients (*n* = 9, blue circles) and healthy controls (*n* = 9, red squares). Data points represent the mean ± standard deviation; vertical dotted lines indicate the ingestion of 100 mL of water or 200 mL of liquid meal.

Phospholipid concentrations were measured in 166 samples from PD patients (83 fasted and 83 fed) and 178 samples from healthy controls (88 fasted and 90 fed). The scatter plots in Figure 3E demonstrate that phospholipid concentrations were highly variable but still clearly lower in fasted-state fluids compared to fed-state fluids in both healthy controls (fasted: 0.65 ± 0.84 mM vs. fed: 3.57 ± 2.37 mM) and PD patients (fasted: 1.14 ± 2.00 mM vs. fed: 3.30 ± 2.80 mM). As can be seen in Figure 8, the average phospholipid concentrations over time did not differ between PD patients and healthy controls, although a slightly higher variability was observed in PD patients.

### 3.6. Bile Salts

Bile salt concentrations and composition were measured in 170 samples from PD patients (85 fasted and 85 fed) and 178 samples from healthy controls (88 fasted and 90 fed). As shown in Figure 3F, total bile salt concentrations were marked by large variability in both fasted- and fed-state fluids. Considering this variability, no difference in total bile salt levels could be observed between PD patients and healthy controls, both in fasted-state fluids (7.64 ± 10.01 mM versus 4.73 ± 5.21 mM, respectively) and in fed-state fluids (12.05 ± 11.17 mM versus 11.00 ± 9.06 mM, respectively).

**Figure 9:**
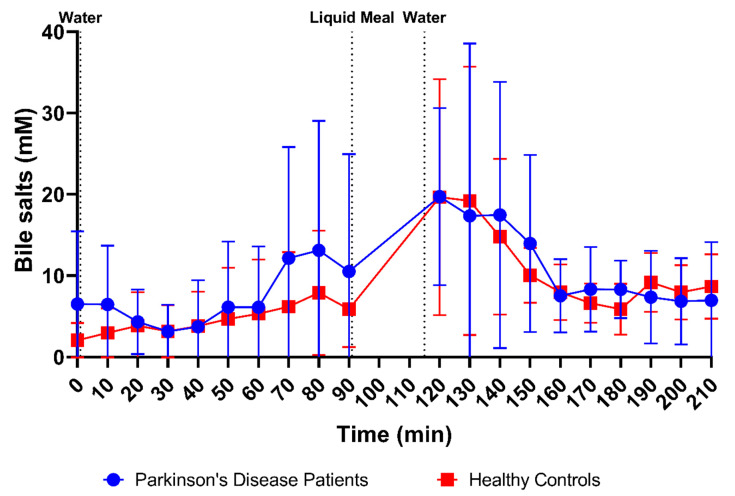
Time-dependent evolution of the total bile salt concentration in duodenal fluids aspirated from Parkinson’s disease patients (*n* = 9, blue circles) and healthy controls (*n* = 9, red squares). Data points represent the mean ± standard deviation; vertical dotted lines indicate the ingestion of 100 mL of water or 200 mL of liquid meal.

In addition, the profiles in Figure 9 depict no differences in the average bile salt concentration over time between PD patients and healthy controls. Despite substantial variability, bile salt levels remained relatively stable in fasted-state conditions. The apparent increase, seen in PD patients after 70 min, resulted from only two patients with a huge spike in their bile salt concentration. Gallbladder contractions during a phase 3 event of the migrating motor complex may have resulted in temporary spikes of high fasted-state bile salt concentrations. Upon intake of the meal, the bile salt concentration temporarily increased in both study populations (plateauing around 120 min, or 30 min after meal intake) before returning to values similar to the fasted state (reached at around 160 min, or 70 min after meal intake).

**Figure 10:**
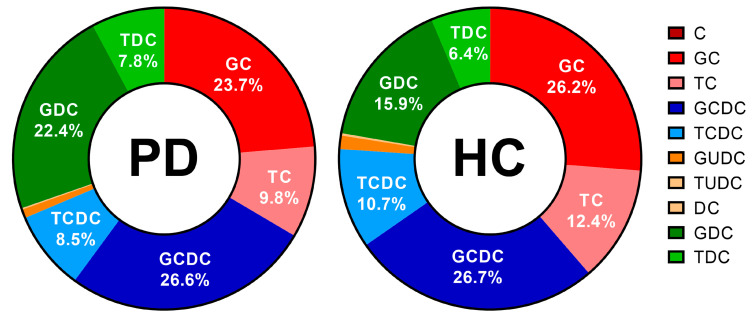
Relative bile salt composition in duodenal fluids aspirated from Parkinson’s disease patients (PD, *n* = 9, left) and healthy controls (HC, *n* = 9, right). Percentages as a function of the total bile salt level were calculated as the average of all time-based samples (i.e., both fasted and fed state) per patient population; percentages of the 6 most abundant bile salts are depicted in the chart. Cholic acid (C), glycocholic acid (GC), taurocholic acid (TC), chenodeoxycholic acid (CDC), glycochenodeoxycholic acid (GCDC), taurochenodeoxycholic acid (TCDC), glycoursodeoxycholic acid (GUDC), tauroursodeoxycholic acid (TUDC), deoxycholic acid (DC), glycodeoxycholic acid (GDC) and taurodeoxycholic acid (TDC).

**Figure 11:**
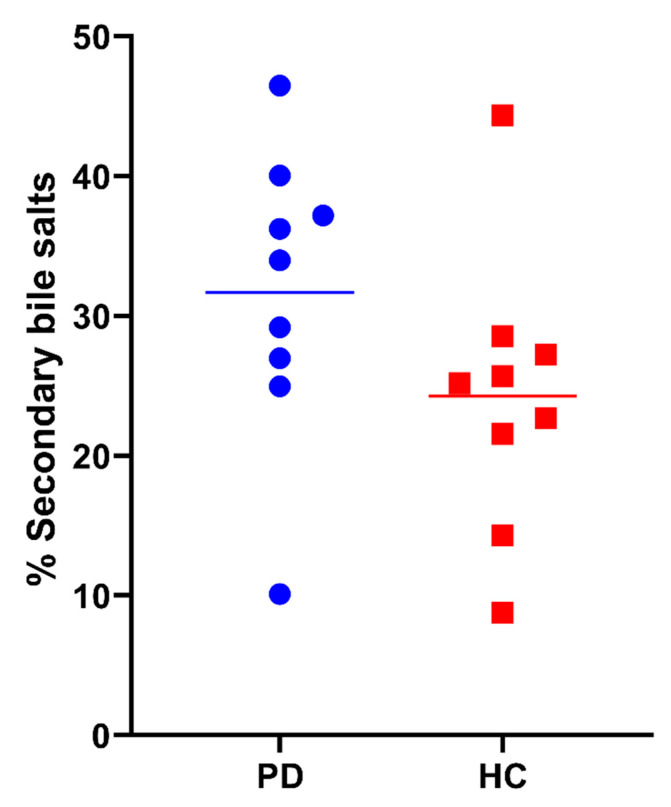
Percentage of secondary bile salts (i.e., GDC, TDC, DC, GUDC, TUDC, UDC and LC) in duodenal fluids aspirated from Parkinson’s disease patients (PD, blue circles) and healthy controls (HC, red squares). Data points represent the mean per subject; lines depict the overall average per study population.

Besides the total bile salt concentration, the relative contribution of different bile salt species was determined. In each subject, the bile salt composition remained stable, independently of total bile salt concentration and prandial state. In Figure 10, the average bile salt composition is depicted for both PD patients and healthy controls. In both study populations, almost all bile salts were conjugated, with the majority being conjugated with glycine (>60%). No difference between PD patients and healthy controls was observed in the rank order of contributing bile salt species (i.e., GCDC > GC > GDC > TC > TCDC > TDC > GUDC). However, the relative amount of secondary bile salts, in particular DC and its conjugates was, in general, higher in PD patients compared with healthy controls, as depicted by the average percentages of secondary bile salts per subject in Figure 11. Different bile salt species may result in different solubilizing capacities and affect the dissolution of drugs in vitro [23,24]. Whether the changes in PD patients also result in differences in solubility and dissolution in vivo requires further investigation.

### 3.7. Lipid Digestion Products and Cholesterol

**Figure 12:**
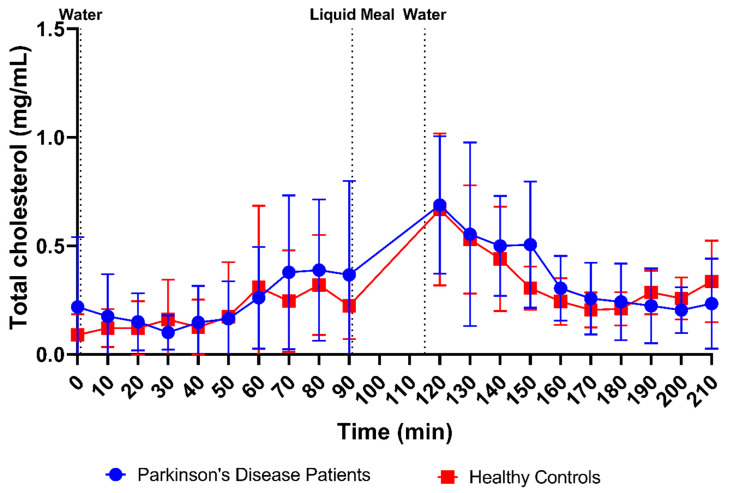
Time-dependent evolution of the total cholesterol concentration in duodenal fluids aspirated from Parkinson’s disease patients (*n* = 9, blue circles) and healthy controls (*n* = 9, red squares). Each data point represents the mean ± standard deviation; the vertical dotted lines indicate the ingestion of 100 mL of water or 200 mL of liquid meal.

Total cholesterol (i.e., cholesterol and cholesteryl esters) and lipid digestion products (i.e., TAGs, DAGs, MAGs and FFAs) were measured in 169 samples from PD patients (84 fasted and 85 fed) and 178 samples from healthy controls (88 fasted and 90 fed). Scatter plots of total cholesterol and lipid concentrations in all fluids are depicted in Figure 3G,H, respectively. No differences were observed in fasted-state cholesterol levels between PD patients (0.23 ± 0.27 mg/mL) and healthy controls (0.19 ± 0.21 mg/mL). In fed-state fluids, cholesterol levels increased to a similar level in both PD patients (0.37 ± 0.28 mg/mL) and healthy controls (0.35 ± 0.23 mg/mL). In addition, the total cholesterol concentration over time profile in Figure 12 did not differ between PD patients and healthy controls, with a similar evolution as seen for the bile salt concentration (Figure 9).

**Figure 13:**
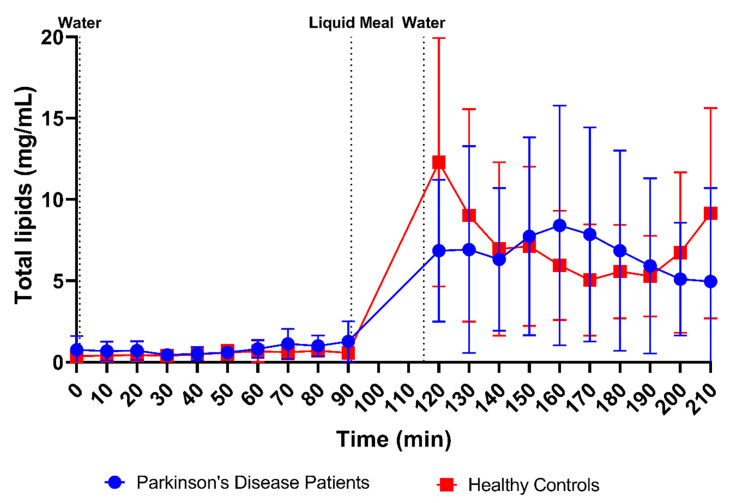
Time-dependent evolution of the total lipid concentration in duodenal fluids aspirated from Parkinson’s disease patients (*n* = 9, blue circles) and healthy controls (*n* = 9, red squares). Data points represent the mean ± standard deviation; vertical dotted lines indicate the ingestion of 100 mL of water or 200 mL of liquid meal.

Although marked by huge variability as shown in Figure 3H, the total lipid concentration also did not seem to differ between PD patients (fasted: 0.78 ± 0.70 mg/mL vs. fed: 6.69 ± 5.48 mg/mL) and healthy controls (fasted: 0.51 ± 0.40 mg/mL vs. fed: 7.31 ± 5.27 mg/mL). The lipid concentration, which was very low in the fasted state, rapidly increased after intake of the meal (Figure 13). Similar to the observations for buffer capacity, osmolality and total protein concentration, the initial meal-induced increase in lipid concentration appeared slower in PD patients compared with healthy controls.

**Figure 14:**
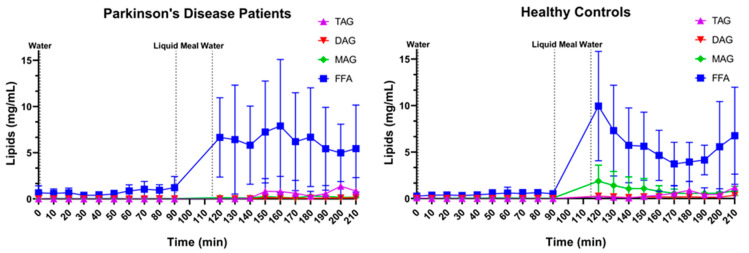
Lipid composition over time in Parkinson’s disease patients (left, *n* = 9) and healthy controls (right, *n* = 9). Data points represent mean concentrations ± standard deviation for the following lipid classes: free fatty acids (FFA, blue squares), monoacylglycerides (MAGs, green diamonds), diacylglycerides (DAGs, red inverted triangle) and triacylglycerides (TAGs, purple triangles). Vertical dotted lines indicate the ingestion of 100 mL of water or 200 mL of liquid meal.

The relative lipid composition over time is depicted in Figure 14 for both PD patients and healthy controls. Almost the entire lipid pool, independent of disease or prandial state, consisted of FFAs. In fed-state fluids, small amounts of TAGs were present, especially in samples with a lower pH (possibly indicating a recent gastric emptying). Surprisingly, relevant concentrations of MAGs were only found in healthy controls but not in PD patients. In PD patients, no MAGs were detected at any of the timepoints, while in healthy controls, up to 15% of the total lipid pool in the first three sampling points after the meal consisted of MAGs.

## 4. Discussion

To our knowledge, this is the first study that investigates the impact of PD on the composition of duodenal fluids. As various GI symptoms, including delayed gastric emptying and microbiome differences, are observed in PD patients, it is not unlikely that the composition of GI fluids would also be affected by the disease. In general, however, all the studied characteristics of fasted- and fed-state duodenal fluids (i.e., pH, buffer capacity, osmolality, total protein, phospholipids, bile salts, lipids and cholesterol) were highly comparable between PD patients and age-matched controls without PD in terms of both average values and observed variability. Moreover, the values reported in the present study with participants between 50 and 78 years old are similar to data from previous duodenal aspiration studies in younger healthy adults (i.e., 18–45 years old) [14,16,19]. This suggests that the composition of duodenal fluids is not markedly altered with age, which has been observed previously for a limited subset of characteristics [25].

Notwithstanding that average values and variability for the various characteristics were highly similar between PD patients and healthy controls, some limited yet noticeable differences were observed in (1) the rate at which certain characteristics changed upon intake of the meal, (2) the relative composition of lipids upon digestion of the meal, and (3) the relative bile salt composition.

Firstly, a slight difference was observed in the time profiles for buffer capacity, osmolality, total protein and lipids. For these characteristics, intake of the liquid meal by healthy controls resulted in a pronounced temporary rise at the first postprandial sampling point (i.e., 30 min post-meal), followed by a decrease. In PD patients, this initial peak was absent; instead, a slower increase was observed, reaching a maximum below the peak level in healthy controls, around 70–80 min post-meal. As all these compositional factors are directly linked to the meal entering the small intestine, their slower change observed in PD patients might be attributed to a delay in gastric emptying. Indeed, a delayed gastric emptying has previously been reported in PD patients, resulting from impaired gastric motility due to the disease [26,27,28]. In addition, the effect may be further enhanced by levodopa, a commonly used drug to treat PD patients, which also delays gastric emptying [28]. For other characteristics, including pH, total bile salts and cholesterol, no such difference in their initial meal-induced change was observed between PD patients and healthy controls. These factors are only indirectly linked to the meal, as they are regulated by feedback mechanisms within the GI tract. Intestinal pH is kept neutral by secretion of bicarbonate into the lumen and responds directly to any changes in intestinal pH; bile salts and cholesterol are secreted by the gallbladder upon stimulation by gastric and intestinal hormones in response to the meal. While cholesterol can also be part of a meal, the liquid meal used in the present study only contained a low concentration of cholesterol (0.02 mg/mL), which is largely negligible compared to the concentrations measured in the duodenal fluids.

While clearly noticeable, the observed differences for certain characteristics between PD patients and healthy controls in response to intake of the meal were rather limited, especially considering the large intersubject variability. However, it is important to realize that the meal used in the present study was in liquid form, while previous studies reported a more pronounced delay in gastric emptying in PD patients receiving a solid meal [29]. Therefore, the effect of delayed gastric emptying on the postprandial composition of duodenal fluids might be higher in PD patients taking a solid meal.

A second difference observed between PD patients and healthy controls involves the lipid composition of the duodenal fluids after intake of the meal. The overall lipid digestion in both PD patients and healthy controls was highly efficient, with the majority of TAGs completely digested into FFAs, even immediately after meal ingestion and in the proximal part of the small intestine. This rapid digestion might be partly explained by the use of a liquid meal. The lipid droplets in the liquid meal are easily accessible by gastric and pancreatic lipases, resulting in rapid digestion in both populations. However, while no MAGs were detected in PD patients, MAGs were found in healthy controls at the first timepoints after meal intake. Further research is needed to elucidate this observation. Possibly, the slower gastric emptying in PD patients results in a more efficient degradation of MAGs, as they are more gradually presented to the intestinal enzymes, while in healthy controls, the initial burst of lipids in the small intestine may temporarily saturate digestion.

Finally, a higher percentage of secondary bile salts, in particular DC and conjugates, was found in the duodenal bile salt pool of PD patients compared with healthy controls. Deoxycholic acid is a secondary bile salt formed by bacterial degradation of primary bile salts (i.e., cholic acid and chenodeoxycholic acid) in the distal small intestine and colon, which are subsequently reabsorbed and transported back to the liver and gallbladder (enterohepatic recirculation). Most likely, the observed differences are therefore linked to alterations in the intestinal microbiome in PD patients, as have been reported previously [30,31]. It should be noted that alternative reasons, including possible differences in the uptake of primary or secondary bile salts in PD patients, cannot be excluded. Besides potentially affecting drug solubility or dissolution, increased secondary bile salts have also been linked to gastroesophageal reflux disease, which is prevalent in PD patients [32,33,34].

## 5. Concluding Remarks

From previous research, it is evident that PD induces changes in gastrointestinal motility and microbiome that may affect processes underlying intestinal drug absorption. The present study indicates no marked effects of PD on the composition of duodenal fluids. Only slight differences were observed between PD patients and controls, which can likely be attributed to the known effects of PD on gastric emptying and the microbiome. Whether these limited differences directly affect oral drug absorption and should be considered when simulating drug absorption in PD patients remains to be investigated. At the very least, the differences may add to the high intersubject variability in intestinal fluid composition, which is evident from every study characterizing these fluids (including the present research). Given this variability, it seems more urgent to provide drug manufacturers with the means to evaluate the sensitivity of drug absorption to changes in GI fluid characteristics [35]. Currently used SIFs, while improving the predictability of in vitro dissolution and permeability tools, lack the ability to incorporate variability [12,36]. The development of a wider range of SIFs and sensitivity analyses by means of physiology-based absorption modeling could aid to address these shortcomings and guide the development of drug formulations that allow robust drug absorption in multiple populations, including PD patients [37].

## Figures and Tables

**Table 1:** Patient demographics.

Patient	Sex	Age	SCOPA-AUT GI	HY Score	Time since Diagnosis (Years)	Medication
PD01	F	64	4	1	1	Levodopa/benserazide, rasagiline, clopidogrel, ezetimibe/simvastatin
PD02	M	66	5	3	2	Levodopa/benserazide, rasagiline, rosuvastatin, levocetirizine, clonazepam
PD03	M	54	5	2	7	Levodopa/benserazide, safinamide, carbamazepine, clonazepam
PD04	M	72	3	1.5	1.5	Rasagiline, pramipexole, rivaroxaban
PD05	M	75	3	1	0.5	Pramipexole, perindopril, nebivolol, rosuvastatin, aspirin
PD06	F	50	7	3	4	Levodopa/benserazide, rasagiline, pramipexole, solifenacin, tramadol, zolpidem
PD07	F	77	8	3	6	Levodopa/benserazide, safinamide, trazodone, mirtazapine, simvastatin, aspirin
PD08	M	78	10	3	10	Levodopa/benserazide, pramipexole, amantadine, selegiline, bisoprolol, tamsulosin, triptorelin, fentanyl, alprazolam
PD09	M	67	4	1.5	7	Levodopa/benserazide, rasagiline, ropinirole
HC01	M	66	0	-	-	Beclometasone/formoterol, mometasone
HC02	F	59	0	-	-	Desloratadine, irbesartan, estradiol/norethisterone acetate
HC03	M	75	1	-	-	Dutasteride/Tamsulosin, rupatadine
HC04	F	52	0	-	-	None
HC05	F	57	0	-	-	None
HC06	F	71	0	-	-	None
HC07	F	61	0	-	-	None
HC08	M	77	0	-	-	Bisoprolol
HC09	M	61	0	-	-	Tamsulosin, allopurinol

PD: Parkinson’s disease patient, HC: age-matched healthy control, SCOPA-AUT GI: Scales for Outcomes in Parkinson’s Disease—Autonomic Dysfunction gastrointestinal section (max 21), HY score: Hoehn–Yahr score ON medication.

## Data Availability

Data will be made available on request.

## Supplementary Materials

The following supporting information can be downloaded at: https://www.mdpi.com/article/10.3390/pharmaceutics15041243/s1, Supplementary Table S1. Analytical gradient for the separation of bile salts, Supplementary Table S2. Mass transitions with collision energy for the individual bile salts, Supplementary Table S3. Analytical gradient for the separation of lipid and cholesterol products, Supplementary Table S4. List of analyzed lipid classes with reference compound and calibration curve range, Supplementary Table S5. Validation results, average accuracy and RSD, combining three separate runs with *n* = 3 per level.
