# Supplementary material for: Characterization of Aspirated Duodenal Fluids from Parkinson’s Disease Patients
Source: Pharmaceutics. 2023 Apr 14;15(4):1243. doi: 10.3390/pharmaceutics15041243 (PMC10145225; doi:10.3390/pharmaceutics15041243)
Supplement: Supplementary file 1 [file pharmaceutics-15-01243-s001.zip › pharmaceutics-2243618-supplementary.pdf]

Characterization of aspirated duodenal fluids from Parkinson's disease patients

Supplementary material

Supplementary Table S1. Analytical gradient for the separation of bile salts.

Time (min)	Flow rate (ml/min)	% A (ACN)	% B (MeOH)	% C (0.2% acetic acid)
0.00	0.450	0	5	95
0.50	0.450	0	5	95
1.50	0.450	13	48	39
4.00	0.450	13	48	39
7.00	0.450	16	48	36
8.90	0.450	18	48	34
9.00	0.450	33	48	19
10.40	0.450	33	48	19
10.50	0.450	0	5	95
12.00	0.450	0	5	95

Supplementary Table S2. Mass transitions with collision energy for the individual bile salts.

Bile salt	Parent Ion	Parent > daughter (m/z)	CE
C	$[M + H - 3H_2O]^+$	355.3 > 145.2	30
GC	$[M - H]^-$	464.35 > 74.1	40
TC	$[M + H - 3H_2O]^+$	462.25 > 337.35	15
CDC	$[M + H - 2H_2O]^+$	357.3 > 95.2	35
GCDC	$[M - H]^-$	448.3 > 74.15	37
TCDC	$[M - H]^-$	498.25 > 80.1	65
UDC	$[M + H - 2H_2O]^+$	357.3 > 95.2	35
GUDC	$[M - H]^-$	448.3 > 74.15	37
TUDC	$[M - H]^-$	498.25 > 80.1	65
DC	$[M + H - 2H_2O]^+$	357.3 > 95.2	35
GDC	$[M - H]^-$	448.3 > 74.15	37
TDC	$[M - H]^-$	498.25 > 80.1	65
LC	$[M - H]^-$	375.35	-

C: Cholic acid, GC: Glycocholic acid, TC: Taurocholic acid, CDC: Chenodeoxycholic acid, GCDC: Glycochenodeoxycholic acid, TCDC: Taurochenodeoxycholic acid, UDC: Ursodeoxycholic acid, GUDC: Glycoursodeoxycholic acid, TUDC: Tauroursodeoxycholic acid, DC: Deoxycholic acid, GDC: Glycodeoxycholic acid, TDC: Taurodeoxycholic acid, LC: Lithocholic acid.

Supplementary Table S3. Analytical gradient for the separation of lipid and cholesterol products.

Time (min)	%C Isooctane	%A Acetone – ethyl acetate (2:1) with 0.1% Acetic acid
0.000	99.5	0.5
0.010	99.5	0.5
0.150	96.5	3.5
3.500	93	7
4.000	93	7
6.000	70	30
8.500	40	60
8.510	99.5	0.5
16.00	99.5	0.5

Supplementary Table S4. List of analyzed lipid classes with reference compound and calibration curve range.

Class	Reference standard	Calibration curve range
Chol	Cholesterol	0.25 mg/mL – 0.0019 mg/mL
CholE	Cholesterol palmitate	0.25 mg/mL – 0.0019 mg/mL
TAGs	Tripalmitin	0.25 mg/mL – 0.0019 mg/mL
DAGs	Dilinolein	0.25 mg/mL – 0.0019 mg/mL
MAGs	Mono-oleate	0.25 mg/mL – 0.0019 mg/mL
FFAs	Oleic acid	1 mg/mL – 0.0078 mg/mL

Supplementary Table S5. Validation results, average accuracy and RSD are combining three separate runs with n=3 per level.

Lipid class	Concentration (mg/mL)	Accuracy (%)	RSD (%)	Recovery in HIF (%)
Chol	0,02	86	5,1	99
	0,25	85	1,6	
	2,50	98	1,4	
CholE	0,02	89	4,2	93
	0,25	90	1,9	
	2,50	99	1,1	
TAGs	0,02	90	6,7	89
	0,25	92	2,5	
	2,50	105	2,3	
DAGs	0,02	85	6,3	87
	0,25	92	2,2	
	2,50	104	1,7	
MAGs	0,02	102	6,9	83
	0,25	77	2,2	
	2,50	82	1,7	
FFAs	0,08	125	4,7	107
	1,00	97	1,4	
	10,00	101	1,4	

Chol: Cholesterol, CholE: Cholesteryl esters, TAGs: Triacylglycerides, DAGs: Diacylglycerides, MAGs: Monoacylglycerides, FFAs: Free fatty acids.
